# Islands in the stream: a qualitative study on the accessibility of mental health care for persons with substance use disorders in Belgium

**DOI:** 10.3389/fpsyt.2024.1344020

**Published:** 2024-07-12

**Authors:** Clara De Ruysscher, Jürgen Magerman, Ilse Goethals, Mégane Chantry, Deborah L. Sinclair, Philippe Delespaul, Jessica De Maeyer, Pablo Nicaise, Wouter Vanderplasschen

**Affiliations:** ^1^ Department of Special Needs Education, Ghent University, Ghent, Belgium; ^2^ EQUALITY//ResearchCollective, HOGENT University of Applied Sciences, Ghent, Belgium; ^3^ Institut de recherche santé et société, Université Catholique de Louvain, Woluwe-Saint-Lambert, Belgium; ^4^ Vakgroep Psychiatrie en Neuropsychologie, Universiteit van Maastricht, Maastricht, Netherlands

**Keywords:** mental health care, substance use disorders, recovery, treatment services, accessibility

## Abstract

**Introduction:**

Persons with substance use disorders (SUD) make up a considerable proportion of mental health care service users worldwide. Since 2010, Belgian mental health care has undergone a nationwide reform (‘Title 107’) aiming to realize a mental health care system that fosters more intensive collaboration, strengthens the cohesion and integration across and between different services, and is more responsive to the support needs of all service users. Although persons with SUD were named as a prioritized target group, how this reform impacted the lives and recovery journeys of persons with SUD remains understudied. This study aims to investigate how persons with SUD, regardless of whether they have co-occurring mental health issues, experience the accessibility of mental health care in light of the ‘Title 107’ reform.

**Methods:**

Data were collected by means of in-depth interviews with a heterogeneous sample of persons with SUD (n=52), recruited from five regional mental health networks in Belgium. In-depth interviews focused on experiences regarding (history of) substance use, accessibility of services and support needs, and were analyzed thematically.

**Results:**

Five dynamic themes came to the fore: fragmentation of care and support, the importance of “really listening”, balancing between treatment-driven and person-centered support, the ambivalent role of peers, and the impact of stigma.

**Discussion:**

Despite the ‘Title 107’ reform, persons with SUD still experience mental health care services as ‘islands in the stream’, pointing to several pressing priorities for future policy and practice development: breaking the vicious cycles of waiting times, organizing relational case management, tackling stigma and centralizing lived experiences, and fostering recovery-promoting collaboration.

## 1 Introduction

It is widely acknowledged that persons with substance use disorders (SUD) account for a considerable proportion of the targeted service user population in mental health care worldwide (1, 2). Although prevalence figures vary, it is estimated that up to 50 percent of persons with mental health problems have concurrent SUD and vice versa (3–5). Today, there is consensus that recovery from SUD is a highly idiosyncratic and complex process impacting multiple life domains, in which health, personal growth, quality of life, inclusive citizenship and social participation are important dimensions of change (6). While there are several pathways to initiate and maintain recovery (both treatment-assisted and unassisted), a range of integrative and multidimensional treatment modalities are generally put forward as the best-suited to meet the heterogeneous needs and support the recovery journeys of persons with SUD (7, 8). Still, however, the treatment coverage of persons with SUD remains poor. A global study by Degenhardt and colleagues showed that only 7.1% of persons with past-year SUD received adequate support in high-income countries, 4.3% in upper-middle-income countries and 1.0% in low-income countries (9).

One key condition for realizing more recovery-oriented, integrative and person-centered support for persons with SUD is intensive collaboration and exchange of expertise between generic mental health care services and specialized addiction treatment services (10, 11). In Belgium, mental health care and specialized addiction treatment have traditionally functioned as two categorically separate sectors. However, from the 1990s onwards, consensus grew to reorganize the mental health field in favor of more integrative care that is more competent and sensitive towards the support needs of specific groups, such as persons with SUD (12). Since 2010, Belgian adult mental health care underwent a nationwide transformation, referred to as the ‘Title 107’ reform, aiming at promoting community-based support, strengthening continuity and integration of care and reducing the long-term uptake of psychiatric hospital beds (13). Through this reform, the Belgian mental health care landscape was divided into 20 regional mental health networks, responsible for providing five care functions: (1) primary mental health care, (2) outreach, (3) social integration and recovery, (4) intensive inpatient care, and (5) long-term residential facilities. These functions are operationalized through pre-existing and newer initiatives, including mobile teams providing mental health care at home, psychosocial rehabilitation centers, intensified short-term residential treatment and supported housing initiatives. One of the core incentives of this reform was de-categorization, i.e. the implementation of collaborative procedures and the strengthening of cohesion between and across different services to supply integrated care across different welfare sectors (14). In the original ‘Title 107’ blueprint, persons with SUD were explicitly named as one of the priority target groups for the mental health care networks. To date, however, no specific action has been undertaken to deliver care tailored to their support needs. Moreover, in 2019, the Belgian Healthcare Knowledge Center raised that there remained several organizational barriers to appropriate mental health care for persons with complex needs, including persons with SUD and co-occurring mental health problems (15). Likewise, international research illustrates that issues relating to poor collaboration within and across the mental health care and specialized addiction treatment sectors persist and reinforce barriers to adequate care (e.g. waiting lists, lack of staff training, stigma) (13, 16).

These challenges are also reflected in a recent WHO report cautioning that the human rights of persons with mental health problems (as stated in the UN Convention of Rights of Persons with Disabilities) remain violated (e.g. in terms of accessibility, discrimination, full participation) despite de-institutionalization reforms globally (17, 18). The growing pains and persistent challenges of nationwide mental health reform significantly and directly affect the micro-level everyday lives, recovery experiences and care trajectories of service users. However, this impact remains understudied, as research focusing on macro-level (i.e. networks) and meso-level (i.e. services and professional stakeholders) evaluations and developments of such reforms have thus far been prioritized (e.g., 19–21).

At the heart of any macro-level mental health reform lies the ambition to positively affect the lives and recovery journeys of individual service users. However, these high-level reforms unintentionally risk reproducing existing barriers and creating new challenges to delivering adequate support (22). To cultivate suitable and efficient strategies for addressing these barriers, it is imperative to gain insight into how they manifest in the everyday lives of service users (23). In the Belgian case, although persons with SUD were explicitly put forward as the target population of the ‘Title 107’ reform, how they experience the accessibility of mental health care has not been investigated since the start of the reform. Addressing challenges in mental health care innovation necessitates bottom-up collaborative (research) practices actively involving (persons with) lived experience (23, 24). This study aims to take a first step in this direction by investigating how persons with SUD, regardless of whether they have co-occurring mental health issues, experience the accessibility of mental health care services in the context of the Belgian reform.

## 2 Methods

### 2.1 Methodological approach

This study aimed to gain insight into the accessibility of mental health care for persons with SUD, by focusing on service users’ experiences. A qualitative methodological framework was employed, and data were collected using in-depth interviews and were analyzed through thematic analysis.

### 2.2 Research location and participants

**Figure 1:**
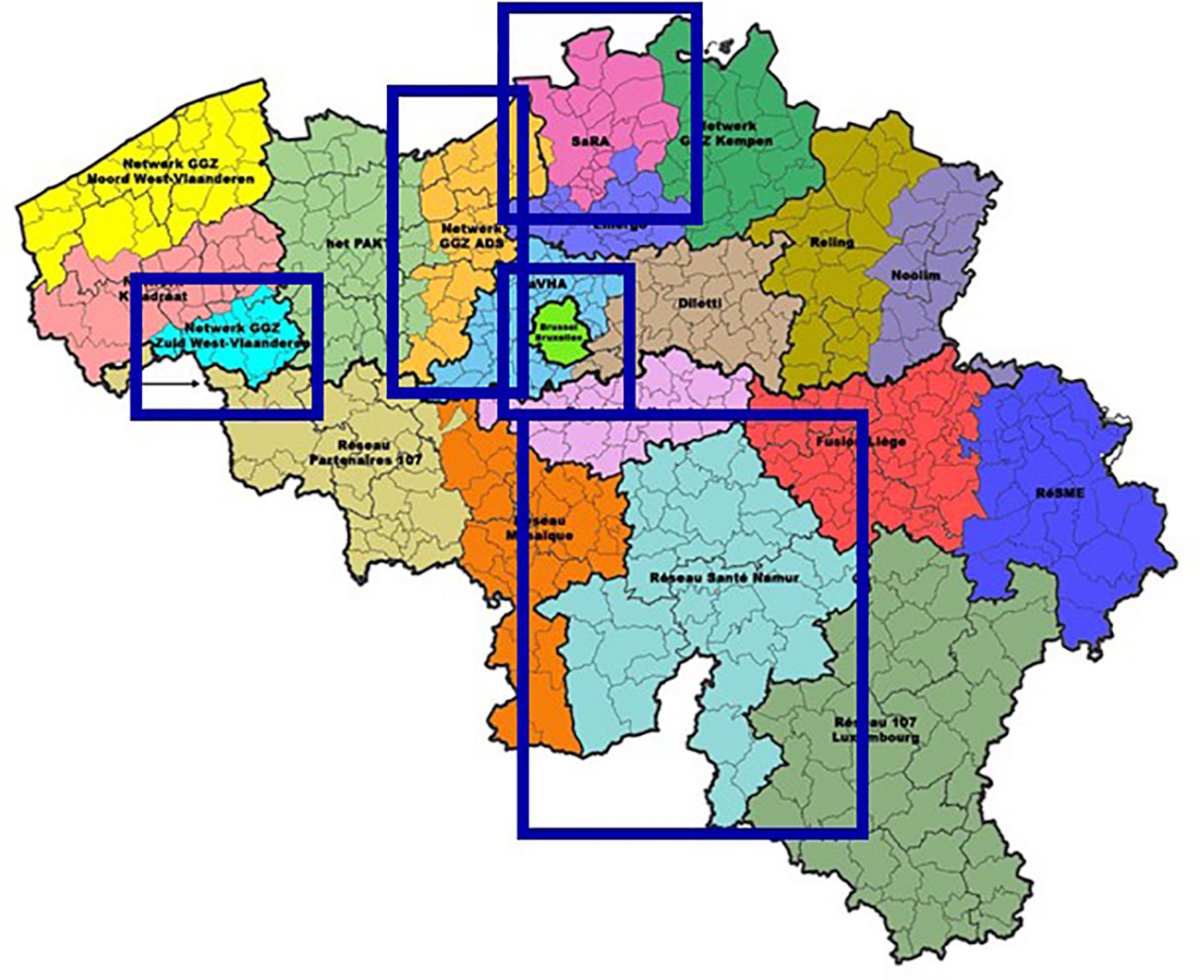
Included mental health networks. Dienst Psychosociale Gezondheidszorg. (2020). https://www.psy107.be/.

Participants were recruited from five different mental health networks in Belgium (3 in Flanders, 1 in Brussels and 1 in Wallonia), as depicted in **Figure 1**. The following inclusion criteria were applied: (1) being at least 18 years of age, (2) having self-reported current or past support needs related to problematic substance use and mental health issues, and (3) being proficient in Dutch or French. The primary focus of this qualitative study was on the accessibility of mental health care for persons with SUD, regardless of whether they have co-occurring mental health issues. While individuals with dual diagnoses may have been included, they were not specifically or exclusively targeted. Intending to obtain a diverse and inclusive sample, the sole exclusion criterion was the presence of acute symptoms of mental illness (e.g. psychotic episode) or withdrawal, and persons who were significantly under the influence of substances at the time of data collection. The invitation for participation was shared with service users by staff members working at mental health care services involved in the five selected mental health care networks (Namur, Brussels, South-West-Flanders, Aalst-Dendermonde-Sint-Niklaas and Antwerp). In the recruitment process, we aimed to obtain diversity regarding gender, age and the extent to which substance use problems impacted several life domains. We also aimed to maximize diversity regarding the type of service used when interviewed, based on the five care functions defined in the mental health reform. Besides active service users, we also recruited persons with SUD who were not followed up by any service to understand their reasons for dropping out. We aimed to reach these persons through low-threshold services (e.g. night shelters, street work services and frontline social services) and snowball sampling. For this specific population, we applied an additional inclusion criterion of not having had contact with mental health or specialized addiction services in the past three years. A single overnight stay in a psychiatric ward was not considered an exclusion criterion. Persons with SUD followed up by low-threshold services could be reached relatively easily because the researchers embedded themselves in the operational functioning of these settings for several days to build trust with the target group. Participants appeared willing to recount their stories, feeling that their experiences were finally being heard. Recruitment of participants from other settings also proceeded smoothly, with participants indicating the importance of the accessibility of appropriate care.

This recruitment strategy led to the inclusion of 52 participants across five mental health networks: 8 in Namur, 11 in Brussels, 9 in South-West-Flanders, 14 in Aalst-Dendermonde-Sint-Niklaas, and 10 in Antwerp. **Table 1** provides an overview of participant characteristics. We have opted not to include specific data on the mental health issues of participants due to this study’s focus on exploring the accessibility of mental health care for persons with SUD, irrespective of co-occurring mental health issues. In doing so, we intended to highlight the heterogeneous and multifaceted experiences and needs of service users with SUD, rather than categorizing them by specific psychiatric problems or diagnoses.

**Table 1:** Participant characteristics.

Characteristic	Categories	N
***Gender***	Male	32
	Female	20
***Age***	[20-29]	7
	[30-39]	14
	[40-49]	17
	[50-59]	6
	[60-69]	7
	Age unknown	1
***Self-reported main type of substance use***	Legal substances (alcohol)	13
	Illegal substances	25
	Mix of illegal and legal substances	14
***Primary care function of the service in which participants were recruited***	Not in contact with services	6
	Primary mental health care	8
	Outreach	11
	Social integration and recovery	7
	Intensive inpatient treatment	13
	Long-term residential facilities	5

### 2.3 Data collection and analysis

Semi-structured in-depth interviews (25) were guided by an interview schedule focused on participants’ experiences regarding their (history of) substance use, past and current use of services, (un)met support needs and helping and hindering factors regarding the accessibility of services. While some participants described their experiences openly, sharing their feelings and emotions, others provided more factual and practical responses, requiring further probing. Interviews (n=52) lasted approximately one hour and were audio-recorded, transcribed verbatim, and analyzed through an inductive thematic approach (26). In the first analysis phase, a subset of seven key interviews was selected based on the richness and diversity of the experiences they captured. The first author (CDR) then conducted an in-depth analysis of each key interview, becoming familiar with the data and assigning initial codes and generating initial themes, represented visually as a mind map with emerging and interconnected superordinate themes (27). This initial thematic structure was discussed in-depth with co-researchers JM, IG, and MC to refine potential themes and sub-themes. Based on this, themes were defined and named (26, 27). In the second analytical phase, this thematic structure was used as a guiding framework for analyzing the remaining interview data, leading to a fine-grained analysis of participants’ experiences. This was then discussed and finalized by the entire research team.

### 2.4 Ethical considerations

This study was approved by the Ethics Committee of Ghent University Hospital (reference number B4032021000133). Participants provided written informed consent before participation in the study and received a 20 euro supermarket voucher as compensation.

## 3 Results

Despite the adapted network structure following the ‘Title 107’ reform, participants still experienced mental health care services as isolated ‘islands in the stream’. This metaphor aptly captures how participants still experienced mental health services as separate and distinct entities despite the reform’s ambition to realize more intensive collaboration and greater cohesion between and across services in generic mental health care and specialized SUD services. In contrast, participants reported encountering several challenges in accessing and navigating these loose networks. More precisely, five main themes emerged from the data: (a) fragmentation of care and support, (b) (lack of) “really listening”, (c) balancing between treatment-driven and person-centered support, (d) the ambivalent role of peers, and (e) stigma. Within each theme, we captured a variety of experiences and ambivalences, confirming the idiosyncratic nature of participants’ needs. We applied a dynamic lens to the facilitators and barriers affecting the accessibility of mental health care for persons with SUD. We did not distinguish between generic mental health services and specialized SUD services in our analysis due to the participants’ heterogeneous treatment experiences. This approach aimed to reflect the complexities and ambiguities in their narratives, thereby highlighting the diverse recovery trajectories within the SUD population. The distinguished sub-themes within each theme aim to capture these ambiguous dynamics.

### 3.1 Fragmentation of care and support

Participants experienced mental health care as a fragmented and dispersed field that is challenging to navigate, influenced by different aspects: the ripple effect of waiting lists and so-called ‘island logic’ within a network structure.

#### 3.1.1 The ripple effect of waiting times

It is a long-standing fact that waiting times are a structural barrier to accessing appropriate services, both within the generic mental health care and specialized addiction treatment system. Waiting times are highly variable and can differ significantly between treatment settings, mental health care regions, and periods. The experiences of the participants allowed us to look beyond this systemic reality and gain an understanding of the rippling effects waiting times caused in the recovery processes of persons with SUD. Several participants explained how the momentum and motivation to seek support lie in moments of crisis, when they have hit rock bottom in one or several life domains. Finding oneself on a waiting list in such a moment of crisis can enhance feelings of desperation and lead to dangerous situations. Participants spoke about how waiting times jeopardized their health because they felt completely alone when physically weaning themselves off drugs:

“The waiting lists are the hardest. You want to quit in that moment. You’ve had enough, you want to stop. But if you then have to wait for three months, then you won’t stop. I tried once to quit on my own, but I ended up in the emergency ward and was nearly dead. So, that wasn’t a good idea. I can only quit using with a lot of support.” (male, age 40-50)

Often, the more specialized and long-term the support provided within a certain service, the longer the waiting time. Especially (long-term) residential support proved to be in high demand. A consequence of these waiting times is that other mental health services, designed to provide *ad hoc* and short-term support, are increasingly used by persons with SUD to “patch up” the gaps created by waiting times in specialized settings. This was particularly the case for psychiatric wards in general hospitals, where the duration of admission is usually limited to up to four weeks. One participant explained how fortunate he was that the generic ward he was admitted to used its discretionary space to allow him to stay for four months:

“Actually I stayed there [psychiatric ward of a general hospital] for so long [4 months] because I was on a waiting list here and I was scared that if I would go home, I wouldn’t make it back here. I used it as a patch-up. Because the year before, they had also suggested to follow a long treatment program and then I went home and didn’t make it. (female, age unknown)

Because psychiatric wards in general hospitals primarily fulfill the function of being a short-term pit stop in space and time, the focus often does not lie on the long-term recovery trajectories of service users, albeit through treatment orientation elsewhere. Moreover, staff are often not specifically trained in supporting persons with SUD. Waiting times also result in adequate support, when it is finally available, no longer being in sync with the recovery trajectories of service users. That is, when support finally becomes available, it risks being mismatched with one’s support needs at that specific time, considerably reducing the chances of a helpful treatment trajectory:

“I’m on the waiting list for seven months now, which is way too long actually. Because you call when you feel bad, not when you feel good. Actually I was okay again already, in terms of my psychosis. Actually I was at work again. And suddenly they called: you can come in. So I take that opportunity because I believe in [facility]. A dual diagnosis ward, not many instances have that. But the waiting list is just terribly long and I can well imagine that many people … drop out.” (male, age 30-40)

Rather than using support modalities that are the best fit with their personal needs and understanding and stage of recovery, waiting times force service users to accept the first available service, whether this is located within generic mental health or specialized addiction treatment services.

#### 3.1.2 Island logic within a network structure

Mental health services are expected to actively provide treatment orientation to partners within their regional network, either as follow-up after treatment or when they cannot provide the most appropriate support themselves (e.g. due to treatment focus, waiting times, black lists). However, participants were often not adequately referred at crucial moments in their recovery process. This contributed to the fragmentation of care trajectories, a lack of motivation, and vicious cycles of problematic substance use. For example, for one participant, fragmented short psychiatric admissions became an inherent part of his recurrent pattern of problematic alcohol use:

“What do you think of psychiatric wards in general hospitals? “That goes really smoothly. Yeah. That is … In less than a week you’re in. But you’re also out quickly again. It is maximum 10 days. (…) It is emergency detox.” And was that helpful to you? “Yeah, you are rid of those withdrawal symptoms for a while.” So when you leave again, do they send you to the social service? “Just back home.” And what did you do at home? “Just start drinking again. (…) That’s how it works, you are sent from one thing to another.” (male, age 40-50)

It is not clear whether these experiences of inadequate referral specifically apply to service users with SUD, or if they point to a more general bottleneck in the ways services collaborate within regional mental health networks. However, the findings affirm that collaboration is essential for continuity of support and how certain services, despite being embedded within a network structure, still apply ‘island logic’ to their daily practice. This bottleneck was also mentioned concerning the bridge between frontline workers and more specialized services. The lack of information and treatment orientation can be demotivating:

“General practitioner? I did not know that system. She didn’t explain anything to me. She just sent me away. (…) She didn’t give me that information. And I really hope that changes in the future. That they don’t just send people away. You see? That is such a shame. Because I was open to recovery. It’s not like they had to force me or so. It’s not like I go there and start making a fuzz. I am open to recovery and still, it’s denied to me. That’s strange.” (female, 40-50)

One participant explained how, after being refused a service he approached, there was a failure to discuss other possible support avenues:

“Then we called [specialized inpatient ward] together to be admitted as a couple. But … We are both in the red there. (…) And they said, ‘ah no, you have been here three times already, I don’t think our way of working works for you. So find another place’. That was it. Not like, go there or go there. No, it was just, no, it’s not going to work here, find somewhere else. Go look on the internet, they said.” (male, age 40-50)

This lack of information was a recurring obstacle in the care trajectories of several participants, who for example described the multitude of treatment options as overwhelming:

“It is just the same with mental health care, there are so many options. But you just don’t know how … You find yourself in some kind of … In a kind of thing that you’ve never … Like a new chapter that you know nothing about, you see? First, you need to know what your rights are and then you can achieve a lot. But you don’t know, you just don’t know.” (female, age 20-30)

Other participants chose not to disclose their substance use-related support needs to their frontline worker (e.g. general practitioner), who could have made referrals to appropriate services within their regional mental health network. Instead, they felt alone in their search for a possible entry point to the mental health care system. One participant explained how he put himself on several waiting lists, based on an elaborate internet search with a friend:

“Yeah, it’s also because of my best friend that I made it here or that I found this treatment place. So she really … We sat in front of the computer together to look up every kind of organization and to call them and … To look what’s the best option. We then made pros and cons, like that organization is better at that, this one is better at that, and then compared. And then decided what to do, what suits me best. She’s a really good friend.” (male, age 20-30)

Another participant believed there was a missed opportunity to spread information to a wider audience regarding support options for persons with SUD. He attributed this lack of information to societal stigma towards SUD:

“You really need to look, on the internet and so on. We had to search really hard … Yeah, you don’t easily find it, support. We really had to look for it. It isn’t addressed enough. I find it should be on the news. Like they show the suicide helpline on the news, it can also be about those kind of things I find. Or on TV or … Like, if you have problems with drugs, this is where you can go.” (male, age 20-30)

However, at the same time, several participants described how a specific professional played an indispensable role throughout their care and recovery trajectories. These professionals provided tailored information, clarified treatment options, ensured consistency and coherence in treatment choices, were reachable both during and outside of crises, and functioned as gatekeepers. Some participants reported that their psychiatrist or general practitioner fulfilled this positive key role, opening doors to new treatment options and guiding toward settings tailored to their needs:

“[My general practitioner] is the only one who knows my whole file. (…) She knew my situation at home, she knew my three children, she knew about the problems with my youngest son, the forced admission, the drug problems, everything. (…) She knew the situation.” (female, age 50-60)

Participants stressed the necessity of someone taking up the role of case manager throughout their support trajectories. However, describing these actors only as case managers might not do justice to the relationships they build with service users. The enthusiastic and passionate tone participants used when talking about these professionals shows how, above all, relational continuity and person-centeredness lie at the heart of these pivotal relationships. One participant described how the continuous proximity and effort of the social worker handling his case gave him a deep feeling of being worthy of care, which was the decisive factor in accepting specialized support:

“The switch came because that staff member from the social service, that woman who you just saw, she stayed on me. And she signed me up to … How do you call that? Forced admission. She just pulled me out from the pit and put me in forced admission. That’s what really woke me up. That woman cared so much about me to save me like that. And yeah, that was the decisive thing for me, like this is enough, I want to step out. (…) The things she explains to me, I actually should have learned from my parents and from school.” (female, age 40-50)

### 3.2 (Lack of) “really listening”

Related to the above, relational continuity came to the fore as an essential aspect in navigating the mental health care landscape in search of appropriate support. Strikingly, participants often described it as “really listening”, emphasizing the importance of authentic therapeutic relationships and trauma-sensitive care.

#### 3.2.1 Authentic therapeutic relationships

The importance of authentic contact with professionals was prominent in participants’ stories and was a determining factor in the success or failure of services used. For one participant, “the human aspect” was more fundamental than personal comfort or the therapeutic program:

“I have to admit that when I came to [name service] and saw the facilities there, I thought, I won’t stay here. (…) But eventually, because the human contact was so good there, also from the nurses… (…) That’s what made me stay. Their facilities are old-fashioned, not much comfort. Horrible. But the human aspect and the character of caregiving and then the tailored therapists and stuff … They were really good.” (female, age 50-60)

Participants stressed that these relationships should be characterized by sincerity, a non-judgmental atmosphere and a dialogical nature. Participants also foregrounded the value of experiencing a sense of commitment, approachability and trust of professionals with whom they ‘clicked’, as illustrated by a participant describing his bond with a psychiatrist:

“There was one psychiatrist in particular who followed me for several years. She was truly magical. Without knowing it, we followed each other in different hospitals, but each time I found her again and so a bond was created. When I saw her for the first time, she was still an assistant, so it was really … I was kind of her first patient. There was a real bond that had been built up with her, and she was also the first psychiatrist my parents felt comfortable with.” (female, age 20-30)

Relational continuity has a strong enhancing effect over time and across different support settings. However, certain factors prevent such dialogical and authentic relationships from developing. One that stood out was the unequal power dynamics between professionals and service users that are unavoidably at play within treatment settings. This power imbalance was most pronounced in contacts with psychiatrists, in which symptomatology sometimes prevailed and stigmatizing attitudes on substance use shone through:

“I’m dealing with a psychiatrist. Apart from neuroleptics, which I don’t know anything about, I know more than he does about the products they spend all day prescribing. I have more expertise and yet he infantilizes me as a drug addict, even though I have quite a broad expertise.” (female, age 40-50)

The results also showed how lasting therapeutic relationships, for example through long-term outpatient support provided by psychologists, were often jeopardized by a structural financial barrier to the continuity of affordable support. Several participants recalled positive and impactful experiences regarding their relationship with their psychologist. At the same time, some participants explained how they would benefit from continued long-term regular contact with their psychologist, but simply could not afford it:

“I know I need help for the rest of my life. But I also know that I need to pay for it myself. (…) If I say I need my therapist once every three weeks in order to keep functioning, then I should do that. But if I need to pay for it all myself, then I know that one day sooner or later I’ll feel quite good and think I don’t need it anymore. But from the past I know that there will be moments then that I do need it and then it’s too late.” (female, age 50-60)

#### 3.2.2 Trauma-sensitive care and support

Participants often mentioned how authentic therapeutic relationships could only be developed when professionals looked beyond the behavioral aspects of their SUD. Particularly, they referred to the importance of addressing the root causes of their SUD, such as adverse childhood experiences, detrimental social circumstances and trauma. One participant explained how the active acknowledgment of these underlying factors unlocked a new phase in his recovery process:

“Here, in [specialized addiction treatment ward], it was really good to just focus on what it is and then to deal with what’s behind it. (…) Here, I talk with therapist X, and from the start … I hate her. In the sense of … She knows it. She sees through me and she just gets it. (…) Here, I don’t know … Yeah, I really learned how to feel. And that is not easy.” (female, age 20-30)

In some cases, the provided therapeutic activities simply fell short of bringing these underlying dynamics to the fore. One participant reflected on how her traumatic experiences themselves made it impossible for her to take the initiative to talk about them. At the same time, paradoxically, she was aware that addressing them was a necessary part of her recovery process. During her admission, staff members seemed oblivious to this need and the therapeutic activities fell short in eliciting these traumas:

“Now I know, if you have an addiction problem, you need to focus on things in the head. (…) In psychiatry I had the feeling, if I can’t open up myself because I’ve been through so many traumas … You are the professional, you should be able to help me unravel things. That’s what I think now. But back then, it just wasn’t there. I’ve missed that. (…) Nobody could know who I am or what happened to me. And what to do. Especially, what to do to get out. (…) My traumas come up, I’m stuck with them. And they are like a whirlwind storming in my head. And I sit there alone with my thoughts.” (female, age 40-50)

### 3.3 Balancing between treatment-driven and person-centered support

Another factor that significantly impacts mental health care accessibility is the extent to which service users experience a good fit between their personal support needs and what certain services have to offer. In this respect, three dimensions stood out: the intake criteria used by services, the expertise of staff regarding SUD, and how recovery was operationalized within services.

#### 3.3.1 The (in)flexibility of intake criteria

Both generic mental health and specialized addiction treatment services often target a specific service user profile, translated into intake criteria acting as gatekeepers to the service. Participants reported diverse experiences regarding how freely these criteria were applied. One participant experienced how a high level of inflexibility left no room for real dialogue or a person-centered exploration of his needs during intake:

“They just want to hear what they want to hear. Their book says such and such. You have to do it that way and you have to ask that question and if you get an answer, then you send [the service user] walking. It is as if they have been indoctrinated with their [intake] questionnaire in front of them. And if someone answers differently to that [intake] questionnaire, then they are already at a loss. (…) That’s how it comes across to me anyway. (…) And then just like that [they say] ‘Yes, this doesn’t fit with us and we don’t have time for you, good riddance’.” (male, 50-60)

At times, substance use in itself was an explicit exclusion criterion in generic mental health services. One participant was denied access to sheltered housing, which directly contributed to a cycle of substance use and possible relapse:

“I’ve still been turned down for sheltered housing because they know I’m from [service name] and that I’m a consumer. They told me it’s not going to be possible.” And what solutions did they propose? “Follow-up for drug use. Basically, you have to stay on the street and monitor your drug use. It’s a bit complicated though. (…) Because when you have a roof over your head, it’s easier to stop using or to set up a follow-up system. When you’re on the street, what do you do? You just want to use because you’re not feeling well.” (male, age 40-50)

Other participants experienced how some mental health services denied access to persons receiving (opioid) substitution treatment. Whereas this might be related to the service’s perceiving it as a transgression of its substance use policy, it was often described as stigmatizing. For one participant, this barrier significantly obstructed his recovery process:

“I would just like to get in somewhere. (…) And then I’m 100% sure that I can hold on for another year. Or longer. And preferably for the rest of my life, my liver isn’t doing so well anymore. (…) But I’m telling you, in those [generic short-term mental health wards], because I take Suboxone…” That makes that you don’t get access to several services that could help you? “Not a single one. (…) As soon as you mention that you take Suboxone or you’re in a drug rush … No, then you can go home.” (male, age 40-50)

Support modalities rooted in a harm reduction approach and more treatment-oriented support could simultaneously play a valuable role in one’s recovery process. However, from a service intake perspective, these seem to mutually exclude each other.

#### 3.3.2 Training and expertise of staff

Participants’ feeling of having ended up in the ‘right’ or ‘wrong’ kind of treatment was also dependent on the extent to which care professionals had specific expertise regarding SUD. Insufficient training about SUD among staff members enabled some participants to hide or ‘separate’ their SUD, which had differing effects. On the one hand, hiding their substance use problems increased access to generic mental health services. On the other hand, the support they received in these places was not sufficiently tailored to their specific needs. At times, this ‘separation’ strategy even led to misdiagnosis:

“I came out [of the psychiatric ward] more addicted than when I started. Because actually I was there for the wrong reasons. (…) They also said that they didn’t focus on drugs, so I just kept on using. I came in under influence, they didn’t even notice. That’s really bad, but I shouldn’t joke about it. (…) And also, the psychiatrist there, I found it so striking, because … They gave me diagnoses that actually didn’t apply at all. For example, bipolar disorder. Eventually it all turned out not to be true, but I did get medication [benzodiazepines] for it.” (female, age 20-30)

Some participants also reported that staff members in generic mental health care were at times not sensitive enough towards the addiction-related vulnerabilities they experienced regarding prescription medication. Other participants reported that frontline healthcare and social professionals (e.g. general practitioners and social workers) were insufficiently aware of how their attitudes and actions might have a directly negative effect on their recovery process. For example, for one participant, failure to keep an appointment with the social worker was used as a reason for the withdrawal of social benefits, which significantly worsened her substance use problems and led to a feeling of not being supported:

“[The social worker] already docked my pay twice because I didn’t keep an appointment. But when you’re in this (substance use), sometimes you forget the days, so you’re already in a bad way and they dock your pay twice a year.” (female, age 50-60)

Participants often experienced a greater sense of belonging and a better alignment with their long-term and recovery-oriented support needs if services were specialized for persons with SUD (and co-occurring mental health problems).

#### 3.3.3 Operationalizations of recovery

Participants valued a good fit between their understanding of recovery and the way recovery was operationalized in the service they used. In particular, the extent to which abstinence is considered a core condition of recovery was often stressed. For example, one participant saw abstinence as the fundamental starting point of her recovery trajectory. Ending up in a women’s group where using substances was tolerated, was not well-aligned with her vision of recovery:

“I’m in a women’s group … An ex-addicted women’s group. It’s a women’s group for women of the street. But using is allowed there. So the idea is to allow people who use and to support them like that. But I want to quit completely. So I want to take some distance of that women’s group, because when I go there, I see those people stoned. That weighs heavily on me.” (female, age 40-50)

In the same vein, some participants experienced they could not work on their recovery trajectory in services where recovery was operationalized through a strict (hierarchic) structure with many rules. Another recovery-related influencing factor was the extent to which services provide support in all life domains, not just the clinical and functional aspects of recovery. One participant received help with his social problems during admission, which exceeded his expectations and positively impacted retention:

“I immediately noticed how the social department was involved to find out how they could help me. I didn’t have a health insurance, I had nothing. Nothing. And they immediately tried to support me in all these aspects … And then it was continued here in [residential ward], also with the social department and … They really supported me and helped me find solutions. Something I hadn’t expected. I thought, I’m here now and I’ll get sober and I’ll be on my own for everything else. But that wasn’t the case.” (female, age unknown)

### 3.4 The ambivalent role of peers

Peers played a unique and influential role in facilitating access to services, both through their formal presence in services (e.g. as peer workers and service users) and informally.

#### 3.4.1 Identification with peers

Several participants talked about how the presence of peer workers in mental health services was supportive and motivating, as they were assigned a special position with a positive influence. This was mainly attributed to the fact that peer workers, because of their experiential expertise, were able to understand what they were going through and did not have a judgmental attitude towards substance use.

“[The peer worker] sees through you. (…) She just really knows it, she really knows it. She can really look at you and whatever you say, she can really laugh and then inside you feel like ‘oh fuck, she got me’. The staff is good to support you, but peer workers are good to really give you insight. Because also, you just believe them, they know what they’re talking about.” (female, age 20-30)

Another important aspect is the extent to which participants identify with the service user population in available services. While some participants have difficulties identifying with the label of having mental health problems, others would rather be associated with mental health services than specialized addiction treatment services. For some participants, encountering persons with (severe) mental health problems in mental health services had an estranging and even traumatic effect. Other participants mentioned how fellow service users can contribute to feelings of belonging and safety within treatment settings, positively affecting retention. At the same time, participants reported how a lack of identification with the mental health problems or lifeworld of fellow service users can cause feelings of unsafety, leading to drop-out or even *a priori* avoidance of these services.

#### 3.4.2 Word of mouth

Together with the presence of peers and peer workers in mental health care, it became clear how the informal influence of peers was even stronger. Several participants mentioned the role of peers in their own near (e.g. family or close friends) or distant (e.g. people from the same neighborhood) social network who had lived experience with generic mental health care and/or specialized substance use treatment. Informally sharing these experiences between peers appears to be common and acts as a powerful testimony, placing services in an attractive or unattractive light depending on the experiences. Additionally, for some participants, this insider information functioned as the primary source of information regarding the daily practice, characteristics, and approach of services, based on which participants decided whether or not to use the service.

“I’ve been in other admissions where I was together with people who had been in [residential specialized service]. And [residential specialized service] has got a really strong regime. Actually, I was allowed there, but I refused it.” Do you think it wouldn’t be for you? In an [residential specialized service] ward or admission? “Of course it would. Because I have an addiction too. (…) But those rules … You can’t have your phone. You can’t have that. I’ve only heard this from other people of course. But those people have been there so they won’t lie about it.” (male, age 30-40)

The above insights illustrate how peers play an ambiguous role in the accessibility of mental health care services for persons with SUD.

### 3.5 Stigma

Stigma was a recurrent theme throughout the interviews and is entangled with other themes. Three different stigma-related dimensions were distinguished: stigma within mental health care, ambivalence towards labels and stigma within people’s social networks.

#### 3.5.1 Stigma within support and care

Stigma is subtly present within the mental health care system itself, having diverse effects on how participants experience and use available services. Participants’ accounts showed how stigma comes to the fore in multifaceted ways, such as judgmental attitudes, language use, preconceived approaches to treatment planning, and engrained institutional logic. Several participants had mixed experiences with psychologists, especially in outpatient (private practice) settings. Whilst some participants found a lasting and supportive connection with their psychologist, others spoke about how perceived stigma and stereotypical perspectives hampered relational continuity and the possibility of openly talking about substance use. Such relational dynamics might even trigger or reinforce feelings of shame:

“I’ve had certain psychologists who … With whom I felt judged. It was just a kind of vibe of … I had the feeling that they thought ‘yeah yeah, it’s no good…’ And when I had drunk, I made stupid mistakes, adultery, things I would never do when sober so I felt a bit judged. I also tried several ones.” (female, age 30-40)

In certain mental health care settings, the narrow idea of recovery as a linear and abstinence-based process was still dominant. In reality, the recovery processes of service users with SUD often have an unpredictable and slow course, inherently characterized by ups and downs and relapse, challenging the attainment of this abstinence-based norm. Furthermore, anticipated stigma prevented participants from opening up about their SUD to frontline workers. Some participants had developed strategies to compartmentalize these support needs, as in this interaction with a counselor:

“They help me with my social benefits. And yeah, I can always talk to them if something’s wrong. But like [my friend] just said, not about drugs. That’s just for the MSOC. (…) Because I want to keep that separate. (…) I have the feeling they would look at me differently then. Yeah, it’s just a feeling. (…) They would automatically behave differently towards us than we’re used to. Automatically. Whether they want to or not, they would do so.” (male, 50-60)

#### 3.5.2 Ambivalence towards labels

Participants had ambiguous relationships with psychiatric and substance use-related labels. Some participants struggled to identify themselves as someone with an SUD and rather considered themselves as someone with mental health problems. Whilst this reluctance to associate themselves with their problematic substance use in favor of a psychiatric diagnosis lowered the threshold to generic mental health services, it was often rooted in dynamics of self-stigma:

“I never labeled myself as an alcoholic. You can’t tell me I’m an alcoholic. So, I don’t agree with that. Well, I know I am, but I don’t want to know.” (female, age 50-60)

For some participants, it was not so much self-stigma that was at play, but rather their stereotypical ideas about persons with SUD that seemed too far removed from their own lived experiences:

“I find it a difficult topic. I don’t want to be ‘the addict’. In my head I still see an addict as someone sitting in a squat with a needle in their arm, lying on the ground. And it’s not like that at all. I always kept on working, I never had unemployment benefits, I had benefits for just two months. I’ve always worked and I’ve always used. I have a daughter, I also didn’t use in front of her.” (male, age 30-40)

Another participant expressed how he experienced the medical-social center (i.e. a place for harm reduction support) as a risky place to hang out, because of the presence of other persons with SUD:

“Yeah, they are willing to steal from you there. And many of them quietly come and get their medication. But more than half of them come there to do criminal activities. And people like me are easy to rip off. See?” (female, age 40-50)

Other participants had opposing experiences with labels, as they identified themselves as someone with SUD but preferred not to be associated with psychiatric labels. These perspectives were colored by stereotypical ideas about the daily practice of mental health services, raising the threshold to using services situated within the ‘psychiatric’ support landscape. This possibly points to a lack of (access to) correct information about mental health care for persons with SUD:

“Everything related to psychiatry and … I see like … yeah … crazy people. So I can’t imagine that I would do that. And I never had a depression before in my life.” (male, age 30-40)

From a care perspective, labels open doors to specific forms of professional support that might be able to offer person-centered care tailored to one’s needs. From a service user perspective, however, stigma in all its forms has a powerful threshold-raising effect. The notion that once you get a label, you can never get rid of it, also shone through.

#### 3.5.3 Stigma within the own social network

Participants also spoke about the hampering effect of stigmatizing perceptions of SUD and/or mental health problems within their social network:

“Like my mother, she thinks it’s a crazy house here, while there are normal people here, like you and me. (…) It was especially difficult, for my work and family, to say ‘this is what’s going on’. I then also said ‘I have psychosis’. Because I find it sounds less bad than ‘I have an addiction’. (…) People have a really bad idea of what addiction is. Or a mental illness.” (male, age 30-40)

At the wider community level, stigma also influenced participants’ decision-making processes in seeking access to support. One participant witnessed how community gossip was set in motion after her dentist sought help for his problematic alcohol use, shaping her decision not to use specialized addiction treatment herself:

“The day you say ‘I quit’, that’s when they look at you. ‘Ah yeah, she drinks’. That’s when you get a finger pointing at you from those people. I saw it happening to our dentist, how they treat him. He’s a drunk. But we were all equally big drunks, but he gets that label. That’s why I don’t want to go to an addiction ward.” (female, age 50-60)

## 4 Discussion

This study aimed to discern the lived experiences of persons with SUD regarding the accessibility of mental health care in Belgium. Despite the ‘Title 107’ nationwide mental health reform towards more collaboration and de-categorization, participants still experienced mental health care services as ‘islands in the stream’ within the reformed network structure. Just as islands may vary in size and resources, mental health services differ in terms of accessibility, expertise regarding SUD, the vision of recovery, proximity to other ‘islands’, and infrastructure, amongst other aspects. Participants reported feeling lost within these loose networks, struggling to access the right services at the right time and tailored to their specific substance use-related needs. Below, we address several critical challenges that should be prioritized in future research and policy development to enhance the accessibility of mental health care for persons with SUD.

### 4.1 Breaking the vicious cycles of waiting times

Waiting times jeopardize the accessibility of mental health care for persons with SUD in more complex ways than just ‘standing in line’ for appropriate support. They cause a clogged-up system in which, on the one hand, persons with SUD are not able to access the most appropriate services when they need them. On the other hand, persons who have endured lengthy waiting periods may occupy spaces that are not aligned with their current needs, driven by a sense of desperation to secure any available spot. To unclog these dynamics, it is helpful to build on the recently developed ecosystem theory of mental health care (‘Ecosysteem Mentale Gezondheid’) shaping current mental health care innovations in the Netherlands (28–31). Central to this ecosystem theory is how in well-functioning mental health ecosystems, all involved services and actors have specific characteristics and expertise and fulfill unique and complementary roles. The strength of the ecosystem as a whole thus depends on the extent to which services can take on their core role. However, as described above, lengthy waiting times affect and change the services’ daily practices, put considerable pressure on (the possibility of) symbiotic collaborations, and disrupt the homeostasis of ecosystems, resulting in diffuse networks that are hard to navigate for service users. Moreover, a recent study by Williams and Bretteville-Jensen (2022) revealed how lengthy waiting times have a detrimental impact on service users’ psychological and physical health, have adverse effects on social functioning, heavily jeopardize recovery processes, lead to lower motivation to engage in treatment and result in overall greater severity of illness upon entry to the mental health care system (32). In that respect, one of the central propositions worth adopting from the ecosystem’s vision of mental health care is to avoid that service users, influenced by the way mental health care is organized, perceive one singular treatment modality as perfectly aligned with their support needs and thus worth waiting for. Instead, offering and actively promoting a diverse array of options is crucial, built on the premise that other treatment options might present equally viable alternatives that are immediately available, devoid of waiting times. From that perspective, the key to a well-functioning mental health care system lies in offering recurrent options rather than in focusing on one-time interventions, acknowledging that sustained success is not magically guaranteed. At the same time, tackling (the ripple effects of) waiting times remains a wicked problem that requires urgent action from high-level actors across several policy domains, transcending the level of individual services and even the level of mental health networks as a whole.

### 4.2 Organizing relational case management

Positive experiences of participants were almost always related to the continuous support of a key figure (e.g. general practitioner, psychiatrist, social counselor) across different services and stages of recovery, providing person-centered support (“they know me”), strengthening relational continuity and informally taking on the role of case manager (33). In the original ‘Title 107’ blueprint, the principle of case management was foreseen to be the responsibility of the mobile teams. This idea is in line with international de-institutionalization trends, in which case management has generally been allocated to Assertive Community Treatment (ACT) (34, 35). However, thus far, it has not been fully or structurally operationalized, as the ACT model has not been evenly rolled out in all the networks. As a result, several mobile teams do not work according to ACT principles. Moreover, several mobile teams are reluctant to support persons with SUD or to include a professional with substance use-specific expertise in their team. Alongside these operational flaws, a more fundamental question that arises is whether it is possible to structurally roll out a form of case management that guarantees relational continuity for all service users (e.g. by appointing each service user to a case manager). Another question to address is whether it is desirable to organize case management as a separate profession within the mental health care networks. The positive key actors in the participants’ accounts were always actively involved in actual care provision and considered case management to be an inherent part of their job. In short, while providing relational continuity can contribute to the accessibility of mental health care for persons with SUD, we believe this should be a collective responsibility of the network, instead of being allocated to individual case managers. They might risk being burdened with the challenging and unattainable duty of both bridging between different service providers in a fragmented care landscape and providing relational continuity to service users. Such a team-based approach could improve continuity of care and facilitate shared decision-making responsibilities (36), which may diminish the risk of burnout among staff (37). On the other hand, such an approach might increase the complexity of organizing care coordination and communication in a complex healthcare system (36). While case management has been proven to strengthen treatment linking and retention for persons with SUD (38), research has also shown that implementing case management is in itself no guarantee of better relational continuity (39, 40). However, to strive for maximization of relational continuity and case management for all service users, and particularly for persons with SUD, the Belgian system might benefit from structurally integrating a Flexible Assertive Community Treatment (FACT) approach, in which principles of flexibility and continuity are combined to ensure that support is person-centered and to prevent service users from being transferred to different teams when their level of needs change (41).

### 4.3 Tackling stigma and centralizing lived experience

Persons with SUD are among the most stigmatized groups in society. There is ample research showing that this stigma significantly interferes with help-seeking behavior in complex and far-reaching ways (42). Alarmingly, our study affirms how stigma also carries over into mental health service provision through judgmental attitudes and language used by service providers and through institutionalized practices and policies, causing iatrogenic harm. Following the Convention on the Rights of Persons with Disabilities, using SUD as an exclusion criterion to generic mental health care undermines the safeguarding of service users’ human rights, as such vulnerabilities should never be an incentive for exclusion from regular care (43). To enhance the accessibility of mental health care for persons with SUD, actively challenging and counteracting engrained stereotypical ideas and stigmatizing practices within mental health services is of utmost priority (44). In that respect, our findings point towards two realistic frontiers. The first challenge relates to the ways psychiatric diagnoses and substance use-related labels function as gatekeepers or barriers to mental health services. Our study showed how service users relate to these labels in highly ambivalent ways, often due to self-stigma, impacting the accessibility of mental health care services (45). While diagnostic labels can facilitate access to services, they can also have the adverse effect of raising the threshold of the same services, as service users are required to actively and openly identify with these labels to gain entry. These struggles often remain under the radar of service providers but have a profound effect on how service users navigate their care trajectories. A greater sensitivity of frontline workers and mental health care providers towards these ambiguous relationships with labels is warranted. In that respect, both sensitivity training aimed at reducing stigma and specific training of service providers regarding SUD might enhance their confidence in working with persons with SUD and would lead to better health outcomes for service users. A second frontier relates to the fact that, despite the increasing deployment of peer workers in mental health care, the representation of peer workers with lived experience of SUD in generic mental health care services remains low (46). Involving peer workers in service delivery has an empowering effect, as it helps service users overcome self-stigma and foster feelings of hope (47). At the same time, peer workers contribute important expertise regarding (recovery from) SUD to mental health teams, provided that they are given an equal position of “partners in co-creation” of recovery-oriented support (48).

### 4.4 Fostering recovery-promoting collaborations

Recovery is often put forward as a bridging framework to foster collaborations between generic mental health care and specialized addiction treatment services, especially in favor of persons with co-occurring mental health issues and SUD (49). Additionally, there is consensus that recovery processes are highly idiosyncratic in nature and are defined in multiple and multidimensional ways (e.g. abstinence, improved health and well-being, taking up socially valued roles), translated into various possible pathways to recovery (8). However, our study demonstrates how generic mental health care services often continue to endorse narrow views of addiction recovery, promoting sustained abstinence as the only viable recovery pathway. Such narrow views do not bridge but instead divide the mental health care landscape, as they feed into the assumption that substance use problems are the fundamental issue that needs to be tackled before mental health can be addressed, an outdated sequential treatment concept deemed ineffective and failing to recognize the importance of addressing trauma in supporting persons with SUD (50, 51). In contrast, persons in recovery benefit from integrated treatment systems in which different types of generic and specialized support are (simultaneously) accessible at different points in their recovery process, aligned with their evolving support needs (52). Earlier work underscores the importance of promptly accessible integrated treatment services for addressing mental health and substance use (53). This contrasts with the complex, fractured systems and services operating in silos so frequently encountered by service users (54). To operationalize such integrative mental health care systems, more productive collaboration between frontline, generic, and specialized services needs to be fostered. Integrated care calls for a fundamental shift towards shared decision-making between all parties involved, including persons with mental health and substance use concerns (54). A cornerstone of service delivery is the concept of ‘no wrong door’, referring here to the delivery of care beyond a specific organization’s boundaries, and facilitating access to other substance use, mental health or other services to ensure all needs are met (55). To operationalize such integrative mental health care systems, more productive collaboration between frontline, generic and specialized services needs to be fostered. This can only be attained through a shared vision of mental health and addiction recovery, in which nuanced and multifaceted meanings of recovery are adopted. Only by actively promoting multiple pathways to recovery (e.g. non-abstinent recovery, controlled use, abstinence-based recovery) can recovery truly act as a bridging philosophy between sectors, enabling more adequate referrals, co-development of support trajectories and a continuous exchange of expertise, thus significantly lowering barriers to adequate support (7, 8, 56).

### 4.5 Limitations

Several limitations of this study should be taken into account. The first set of limitations is related to the use of convenience sampling for participant recruitment, which may have caused selection bias. Although the five included mental health care networks were chosen based on reaching maximal diversity, they may not be fully representative of all 20 Belgian mental health care networks. Additionally, although a concerted effort was made to include persons with SUD who were not in contact with services, they remained a minority. In future research, convenience sampling should be complemented with other sampling techniques to reach and include the experiences of persons with SUD in highly precarious situations. Second, as this study is situated within the specific Belgian mental health care context, shaped by specific local social, cultural and political dynamics, results should be generalized with care to other international mental health care contexts.

## 5 Conclusion

To transform mental health care networks from ‘islands in the stream’ to more cohesive and collaborative ecosystems, the above-described critical points should be seen as priority areas to be addressed in further research and policy development. Before concluding this article, a critical limitation of this study must be emphasized. One of the goals was to centralize the lived experiences of persons with SUD, as we problematized they often remain overlooked in research evaluating the effects of mental health reforms. The chosen qualitative methodology enabled us to build an understanding of how macro-level developments affect the micro-level lives and recovery processes of persons with SUD. However, to advance the mental health care field, we need to take a step further and move away from top-down policy development and mental health care design. Instead, future mental health care innovation and design should be built on co-productive research approaches that rely on persons with SUD as fully equal partners and decision-makers (24, 57).

Overall, the challenges described in this study do not only specifically relate to the accessibility of mental health care for persons with SUD, but are also symptomatic of underlying flaws of the Belgian mental health reform affecting all service users. In that respect, in striving towards well-functioning mental health care ecosystems, persons with SUD should not be treated as a separate or especially complex category of service users, but as a heterogeneous group with equally diverse needs and visions of recovery as all other service users. We hope that our analysis and recommendations lead to actions that positively impact mental health care delivery for all service users, not least for persons with SUD.

## Data availability statement

The raw data supporting the conclusions of this article will be made available by the authors, without undue reservation.

## Ethics statement

The studies involving humans were approved by Ethical Committee of Ghent University Hospital. The studies were conducted in accordance with the local legislation and institutional requirements. The participants provided their written informed consent to participate in this study.

## Author contributions

CD: Conceptualization, Formal analysis, Investigation, Methodology, Writing – original draft, Writing – review & editing. JM: Conceptualization, Formal analysis, Investigation, Methodology, Writing – original draft, Writing – review & editing. IG: Formal analysis, Investigation, Methodology, Writing – review & editing. MC: Formal analysis, Investigation, Methodology, Writing – review & editing. DS: Conceptualization, Writing – review & editing. PD: Conceptualization, Supervision, Writing – review & editing. JD: Conceptualization, Supervision, Writing – review & editing. PN: Conceptualization, Supervision, Writing – review & editing. WV: Conceptualization, Supervision, Writing – review & editing.
